# Prediction of prognosis and immunotherapy response of amino acid metabolism genes in acute myeloid leukemia

**DOI:** 10.3389/fnut.2022.1056648

**Published:** 2022-12-22

**Authors:** Hui Zhou, Fengjuan Wang, Ting Niu

**Affiliations:** ^1^Department of Hematology and Research Laboratory of Hematology, West China Hospital, Sichuan University, Chengdu, Sichuan, China; ^2^Department of Hematology, The First Affiliated Hospital of Zhengzhou University, Zhengzhou, Henan, China

**Keywords:** amino acid metabolism, prognosis, tumor microenvironment, immunotherapy, acute myeloid leukemia

## Abstract

**Background:**

Amino acid (AA) metabolism plays a crucial role in cancer. However, its role in acute myeloid leukemia (AML) is still unavailable. We screened out AA metabolic genes, which related to prognosis, and analyzed their correlation with tumor immune microenvironment in AML.

**Methods:**

We evaluated 472 amino acid metabolism-related genes in 132 AML patients. The predictive risk model was developed according to differentially expressed genes, univariate Cox and LASSO analyses. We validated the risk signature by survival analysis and independence tests. Single-sample gene set enrichment analysis (ssGSEA), tumor immune microenvironment (TME), tumor mutation burden (TMB), functional enrichment, and the IC50 of drugs were assessed to explore the correlations among the risk model, immunity, and drug sensitivity of AML.

**Results:**

Six amino acid metabolism-related genes were confirmed to develop the risk model, including *TRH*, *HNMT*, *TFEB*, *SDSL*, *SLC43A2*, and *SFXN3*. The high-risk subgroup had an immune “hot” phenotype and was related to a poor prognosis. The high-risk group was also associated with more activity of immune cells, such as Tregs, had higher expression of some immune checkpoints, including PD1 and CTLA4, and might be more susceptible to immunotherapy. Xenobiotic metabolism, the reactive oxygen species (ROS) pathway, fatty acid metabolism, JAK/STAT3, and the inflammatory response were active in the high-risk subgroup. Furthermore, the high-risk subgroup was sensitive to sorafenib, selumetinib, and entospletinib. ssGSEA discovered that the processes of glutamine, arginine, tryptophan, cysteine, histidine, L-serine, isoleucine, threonine, tyrosine, and L-phenylalanine metabolism were more active in the high-risk subgroup.

**Conclusion:**

This study revealed that AA metabolism-related genes were correlated with the immune microenvironment of AML patients and could predict the prognosis and immunotherapy response of AML patients.

## Introduction

Acute myeloid leukemia (AML) is one of hematologic malignancies. The myeloid blasts clonally expand in the peripheral blood, bone marrow (BM), and, or other tissues. AML is the most common in adults with acute leukemia. In recent years, the incidence of leukemia has gradually increased (1). In the United States, 20,050 people are estimated to have AML in 2022, and 11,540 patients will die of the disease (1). The global incidence of leukemia increased by 26% from 2006 to 2016 (2). The prognosis of AML was poor, and only 29.5% of patients survived from 2011 to 2017 (3). AML often occurs in elder adults, and approximately 60% of patients are ≥65 years old (3), with a median age at diagnosis between 68 and 71 years (4).

Chemotherapy and allogeneic stem cell transplantation are still the primary therapies for AML (5). However, drug resistance, refractory diseases, and relapse are still challenge in traditional treatment (6). Over the past few years, immunotherapies for AML have undergone considerable development, such as CD33- or CLL-1-specific chimeric antigen receptor (CAR)-T-cell therapy (7, 8) and immune checkpoint inhibitors, including TIM3, CD47, and anti-CD70 (9–11). However, the immunosuppressive tumor microenvironment (TME) reduces the efficacy of immunotherapy (6, 12, 13).

The TME in hematopoietic malignancies, also recognized as the BM microenvironment, lacks of energy sources and biosynthetic materials (14). Amino acids (AAs) is one of the most important energy sources and biosynthetic materials for tumor and immune effector cells (15, 16). Tumor cells usually obtain more AAs from the TME to maintain survival and proliferation (15, 16). However, AA absence in the TME inhibits the proliferation and differentiation of immune effector cells, decreasing their antitumor effects (17, 18). Moreover, tumor-mediated AA metabolism takes part in the formation of the immunosuppressive TME, which includes immunosuppressive cells, such as regulatory T cells (Tregs), myeloid-derived suppressor cells (MDSCs), and M2 macrophages (19–21).

Increasing evidence has discovered that amino acid metabolism participates in the development and progression of AML, and therapies targeting tumor AA metabolism not only inhibit hematological malignancies but can also overcome drug resistance and enhance the efficacy of immunotherapy (22–25). However, present studies are mostly confined to single gene or single amino acid. The comprehensive analysis of multi-amino acid metabolism in AML remains unknown. Therefore, we comprehensively evaluated the association between amino acid metabolism-related genes and the prognosis and immunity of AML.

## Materials and methods

### Data acquisition

We get the RNA-seq and clinic data of 151 AML patient samples from The Cancer Genome Atlas (TCGA) database.^1^ We finally included 132 TCGA-LAML samples when excluded samples without survival time. Moreover, we downloaded the RNA-seq data and clinical data of 91 AML patients from the GSE10358 database as a validation cohort. Additionally, somatic mutation data were also downloaded from the TCGA database.

### Consensus clustering based on amino acid metabolism-related genes

A total of 447 AA metabolism-related genes were retrieved from the Molecular Signature Database (MsigDB^2^) (Supplementary Table 1). A total of 91 AA metabolism-related genes were correlated with the prognosis of AML by univariate Cox regression analysis (Supplementary Table 1). Consensus clustering was performed based on the expression data of the 91 genes by the “ConsensusClusterPlus” package. The number of clusters was identified by the K-means method, and calculated 1,000 iterations (26). We underwent principal component analysis (PCA) and t-distributed stochastic neighbor embedding (t-SNE) analysis to evaluate the distribution of various groups in the constructed model.

### Construction and validation of the predictive risk model

The differentially expressed genes (DEGs) between clusters 1 and 2 were identified by the limma package and filtered by *P*-value < 0.001 and |logFC| > 1. Univariate Cox analysis was used to select prognosis-related genes based on the DEGs. The least absolute shrinkage and selection operator (LASSO) analysis was conducted by the “glmnet” package. The risk score was calculated: $\mathrm{Risk}\,\mathrm{score}=\sum_{i=1}^{n}\left({\mathrm{Expi}}^{*}\,\mathrm{Coefi}\right)$. Coefi and Expi represent the risk coefficient and gene expression, respectively. According to the median risk score, patients were divided into low-risk and high-risk groups, respectively. Kaplan–Meier survival analysis and receiver operating characteristic (ROC) curves were conducted by risk score.

### Development of the prognostic nomogram

We performed univariate and multivariate Cox regression analyses to recognize independent prognostic factors. We built a prognostic nomogram and calculated the concordance index (C-index) of the risk model. The predictive values of 1-, 3-, and 5-year overall survival (OS) rates and the actual results were assessed by the calibration plot. Time-dependent ROC curves were used to predict 1-, 3-, and 5-year OS by the nomogram.

### Tumor immune environment

The ESTIMATE algorithm was applied to conduct the stromal, immune, and ESTIMATE scores (27). Additionally, we performed the CIBERSORT algorithm to quantify the proportions of immune cells in AML samples. The gene expression of AML samples was used to generate scores of 29-type immune cell-, human leukocyte antigen (HLA)-, and checkpoint-related genes for these samples were conducted by single sample gene set enrichment analysis (ssGSEA) with the gsva package.

### Functional enrichment analysis

The enrichment analysis was performed using the R package clusterProfilert. we used R package ‘‘org.Hs.eg.db’’ for gene ontology (GO) annotations and we obtain the latest kyoto encyclopedia of genes and genomes (KEGG) annotations from KEGG rest API.^3^ For Gene set enrichment analysis (GSEA), we downloaded the GSEA software (version 3.0) from GSEA,^4^ and the samples were divided into high-risk and low-risk groups, to evaluate the related pathways and molecular mechanisms. The heterogeneity of various biological processes was explored by Gene Set Variation Analysis (GSVA) enrichment with the “GSVA” package. Hallmark gene sets “h.all.v7.4.symbols.gmt” were acquired from the MSigDB database and were evaluated by GSVA.

Single-sample gene set enrichment analysis was also conducted to assess the amino acid metabolism-related pathway activity between the two risk subgroups. The genes in the amino acid metabolism-related pathways were acquired from the MSigDB (Supplementary Tables 2, 3).

### Tumor mutation burden and drug sensitivity analysis

The mutation annotation format (MAF) was downloaded from the TCGA database and assessed by the R package “maftools.” The tumor mutation burden (TMB) of each patient was also calculated.

The drug sensitivity data containing 198 compounds were acquired from the Drug Sensitivity in Cancer (GDSC2) website^5^ (28). We predicted the half-maximal inhibitory concentration (IC50) of these drugs by using the “oncoPredict” package (29). A parliament plot was plotted to exhibit the drug sensitivity of low- and high-risk groups using the R package “ggpol.”

### Statistical analysis

R software (version 4.2.1) was used to calculated all statistical analyses. The quantitative and qualitative variables are displayed in the mean ± standard deviation and number (ratio%) format, respectively. The Spearman correlation test was used to calculate association coefficients. Log-rank tests were used to plot Kaplan–Meier analysis curves. *T*-tests or Wilcoxon tests were calculated to compare the normally or non-normally distributed quantitative variables between the two subgroups, respectively. To compare the qualitative variables between the two subgroups, chi-square analysis and Fisher’s test were used. *P*-values < 0.05 were considered statistically significant. * *p* < 0.05, ^**^ *p* < 0.01, ^***^ *p* < 0.001, ^****^ *p* < 0.0001, ns, no significant.

## Results

### Construction of two distinct clusters

**FIGURE 1:**
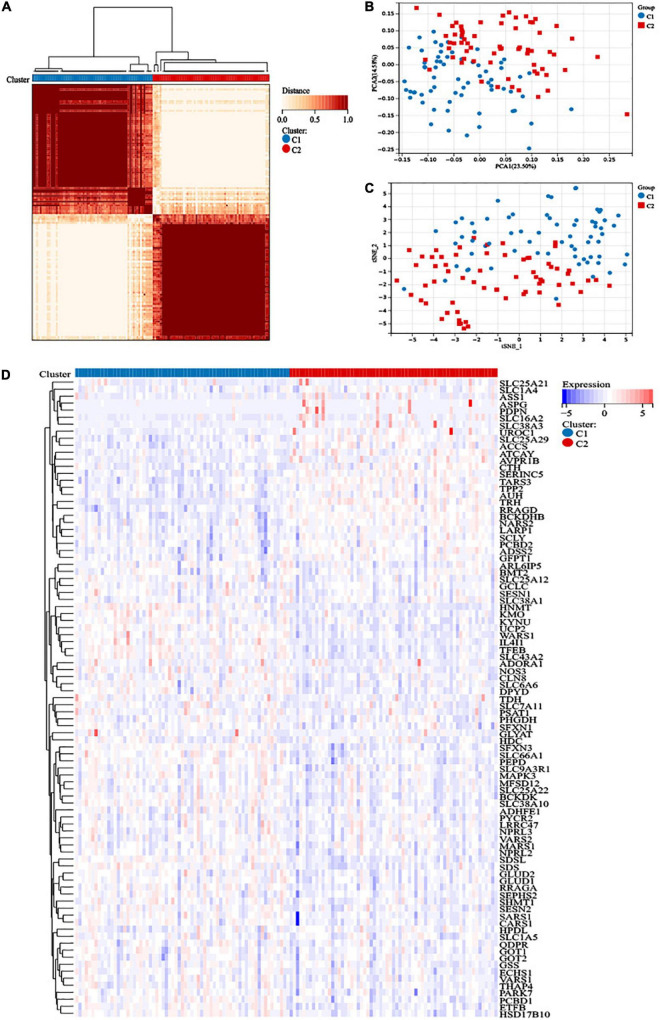
Construction of distinct amino acid metabolism-related clusters. **(A)** The consensus clustering matrix divided 132 acute myeloid leukemia (AML) patients into two clusters (*k* = 2). **(B)** Principal component analysis (PCA) analysis and **(C)** t-distributed stochastic neighbor embedding (t-SNE) analysis showed distribution difference between the two clusters. **(D)** Heatmap of the 91 amino acid metabolism genes in the two clusters.

We obtained a total of 471 amino acid metabolism related genes from MSigDB. Ninety-one survival-related amino acid metabolism genes were further assessed based on univariate Cox regression analysis of 132 AML patients (Supplementary Table 4). A consensus clustering algorithm was used to divide the patients into two clusters according to the 91-gene expression data (Figure 1A). The result was confirmed by the consensus CDF curve, delta area, and optimal number in Nbclust (Supplementary Figures 1A–C). PCA and t-SNE showed different distributions of amino acid metabolism genes between the 2 clusters (Figures 1B, C). Most of the amino acid metabolism genes were significantly upregulated in cluster C1, which indicated relatively active amino acid metabolism in cluster C1. However, cluster C2 showed low expression of most amino acid metabolism-related genes, suggesting decreased amino acid metabolism (Figure 1D).

### Differences in clinical, immunity, and pathway between the two clusters

**FIGURE 2:**
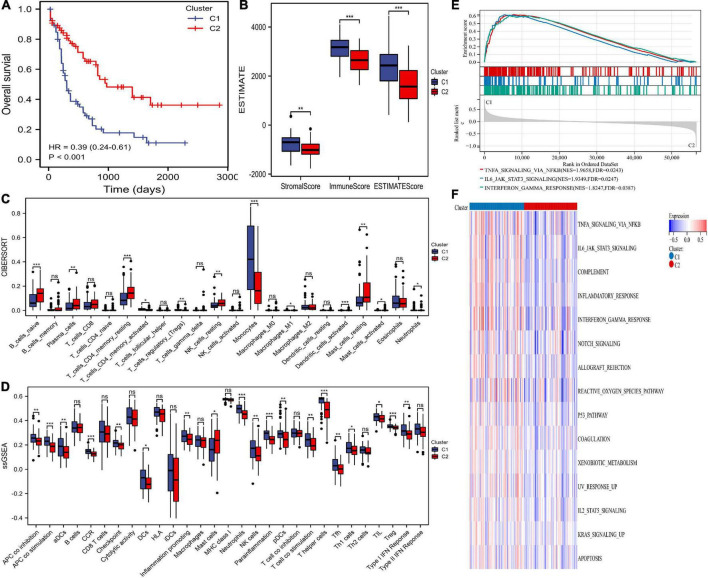
Differences in prognosis, immunity, and pathway enrichment between the two clusters. **(A)** overall survival (OS) of the two clusters. **(B)** The ESTIMATE algorithm calculated stromal, immune, and ESTIMATE scores between the two clusters. **(C,D)** The differences in immune cells and immune responses between the two clusters by the CIBERSORT and single-sample gene set enrichment analysis (ssGSEA) algorithms. **(E,F)** Heatmaps of hallmark analysis in the two clusters by gene set enrichment analysis (GSEA) and gene set variation analysis (GSVA) enrichment analyses. * *p* < 0.05, ** *p* < 0.01, *** *p* < 0.001, ns, no significant.

Survival analysis suggested that cluster C2 had an improved prognosis, while poor overall survival was observed in cluster C1 (Figure 2A). Clinical characteristics, such as age, recurrent gene mutations such as FLT3-ITD, and cytogenetic risk, can cause pathogenesis and progression and impact outcomes. Therefore, we explored the differences in clinical characteristics in the two clusters. We found that patients in cluster C1 showed older age, higher white blood cells (WBC) counts, higher percentages of M4, M5, M6, and M7, advanced cytogenetic risk, and higher frequencies of NPM1 mutations (Table 1).

**TABLE 1:** The characteristics of 132 acute myeloid leukemia (AML) patients between two clusters.

Characteristics	C1 (*N* = 67)	C2 (*N* = 65)	Total (*N* = 132)	*P*-value
Age	57.90 ± 15.34	48.51 ± 16.05	53.27 ± 16.33	0.0008
WBC	38.42 ± 38.64	30.16 ± 45.77	34.38 ± 42.31	0.02
Blast cell (%)	66.79 ± 22.19	64.51 ± 24.29	65.67 ± 23.19	0.73
BM blast cell (%)	34.90 ± 29.94	41.43 ± 32.53	38.11 ± 31.29	0.34
Sex				0.61
Female	29 (21.97%)	32 (24.24%)	61 (46.21%)	
Male	38 (28.79%)	33 (25.00%)	71 (53.79%)	
FAB				8.00E-06
M0	3 (2.27%)	9 (6.82%)	12 (9.09%)	
M1	13 (9.85%)	19 (14.39%)	32 (24.24%)	
M2	15 (11.36%)	17 (12.88%)	32 (24.24%)	
M3	1 (0.76%)	13 (9.85%)	14 (10.61%)	
M4	20 (15.15%)	7 (5.30%)	27 (20.45%)	
M5	12 (9.09%)	0 (0.0e + 0%)	12 (9.09%)	
M6	2 (1.52%)	0 (0.0e + 0%)	2 (1.52%)	
M7	1 (0.76%)	0 (0.0e + 0%)	1 (0.76%)	
Cytogenetics risk				4.50E-06
Favorable	3 (2.27%)	27 (20.45%)	30 (22.73%)	
Intermediate	47 (35.61%)	26 (19.70%)	73 (55.30%)	
Poor	15 (11.36%)	12 (9.09%)	27 (20.45%)	
FLT3				0.28
Negative	42 (31.82%)	49 (37.12%)	91 (68.94%)	
Positive	23 (17.42%)	15 (11.36%)	38 (28.79%)	
NPMc				9.90E-05
Negative	41 (31.06%)	59 (44.70%)	100 (75.76%)	
Positive	26 (19.70%)	5 (3.79%)	31 (23.48%)	

The infiltrating immune cells in different clusters were evaluated. The ESTIMATE algorithm showed that AML patients in cluster 1 had significantly higher stromal, immune, and ESTIMATE scores (Figure 2B). The CIBERSORT algorithm showed that cluster C1 was full of monocytes, M1 macrophages, Tregs, active CD4+ memory T cells, and neutrophils but lacked naïve B cells, plasma cells, resting memory CD4+ T cells, resting NK cells, activated mast cells, and resting mast cells (Figure 2C). Additionally, the ssGSEA algorithm compared 29 immune signatures and suggested that tumor-infiltrating cells, Tregs, checkpoints, inflammation-promoting cells, and APC, HLA, and IFN responses were highly active in cluster C1 (Figure 2D). These results indicated higher immune infiltration among cluster C1, which was consistent the amino acid metabolic activity. However, cluster C2 can be defined as having an immune “cold” phenotype.

We performed GSEA and GSVA of hallmark pathways. GSEA found INFγ, JAK/STAT3, and TNFα inhibition in cluster C2 (Figure 2E). Similarly, signaling pathways such as KRAS, JAK/STAT3, INFγ, and TNFα in cluster C2 were downregulated (Figure 2F).

### Construction and validation of a 6-gene risk model

**FIGURE 3:**
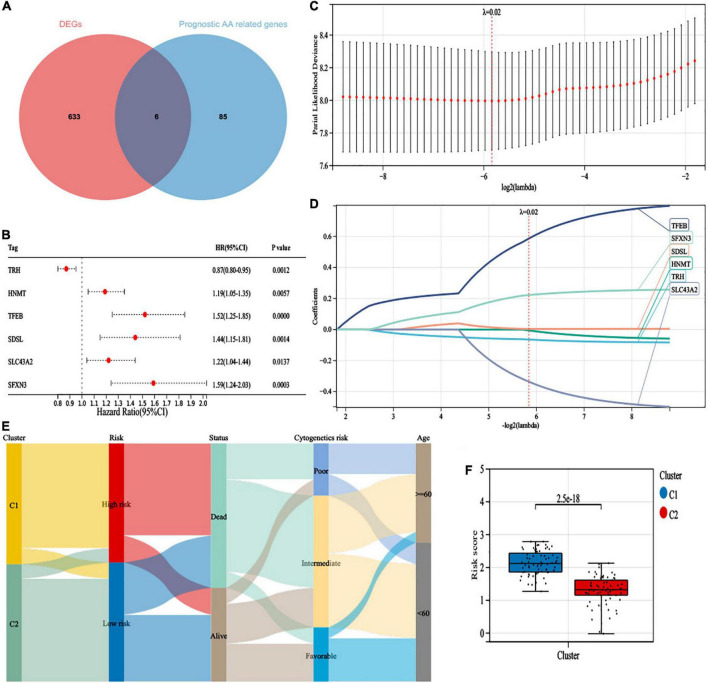
Construction of a 6-gene risk model. **(A)** The 6 survival-related amino acid metabolism differentially expressed genes (DEGs) were developed by a Venn diagram. **(B)** The univariate Cox analysis of OS for 6 survival-related amino acid metabolism DEGs. **(C)** Least absolute shrinkage and selection operator (LASSO) regression of the 6 survival-related amino acid metabolism DEGs. **(D)** Cross-validation for all the 6 survival-related amino acid metabolism DEGs. **(E)** Alluvial diagram of subgroup distributions with different clusters, risk scores, survival status, cytogenetic risk, and age. **(F)** The distribution of risk scores in different clusters.

Differentially expressed gene (DEG) analysis between the two clusters was performed, and 639 DEGs were identified (*P*-value < 0.001, | logFC| > 1), including 431 upregulated genes and 208 downregulated genes. We identified six survival-related amino acid metabolism DEGs (Figure 3A). In addition, *HNMT*, *TFEB*, *SDSL*, *SLC43A2*, and *SFXN3* acted as risk factors, while *TRH* was a favorable factor (Figure 3B). Furthermore, LASSO analysis was conducted to construct the risk signature. Finally, all six genes were included to develop the risk model based on the optimum λ (Figures 3C,D). The risk score was listed in Supplementary Table 5. In addition, to visualize variations in the clusters, risk scores, survival status, cytogenetic risk, and age of AML patients, an alluvial diagram was plotted (Figure 3E). Patients in cluster C1 exhibited higher risk scores; in contrast, cluster C2 had lower risk scores (Figure 3F).

**FIGURE 4:**
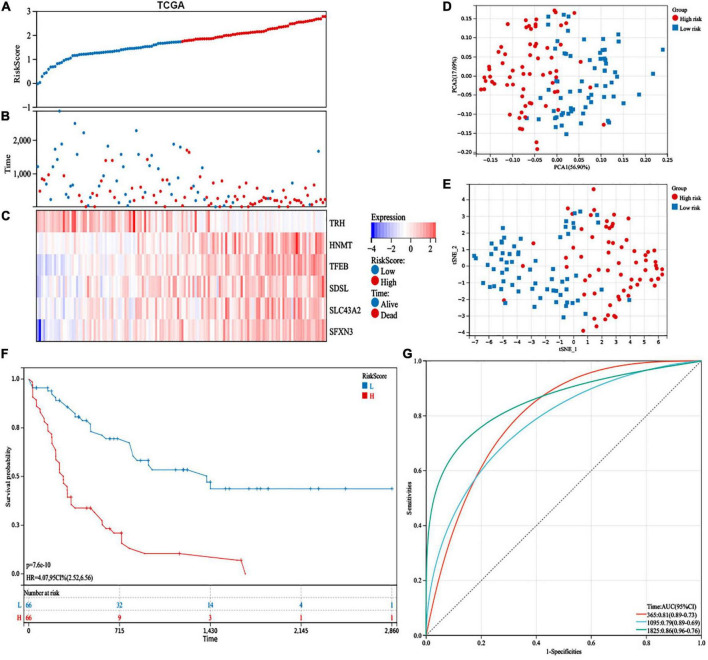
Prognostic study of the cancer genome atlas (TCGA) database in the 6-gene risk model. **(A)** Risk scores, **(B)** survival status, and **(C)** heatmap of the 6 survival-related amino acid metabolism differentially expressed genes (DEGs). **(D)** Principal component analysis (PCA) and **(E)** t-distributed stochastic neighbor embedding (t-SNE) analysis of the six amino acid metabolism-related genes exhibited the differential distribution. **(F)** Kaplan–Meier analysis of OS between the two groups. **(G)** The predictive capability with area under curve (AUCs) at 1-, 3-, and 5-years.

According to the median value, patients were divided into high-risk (*n* = 66) and low-risk (*n* = 66) subgroups (Figure 4A). We indicated that more patients were dead in the high-risk group than the low-risk group (Figure 4B). Additionally, the expression levels of *HNMT*, *TFEB*, *SDSL*, *SLC43A2*, and *SFXN3* were increased, while *TRH* expression was lower in the high-risk group (Figure 4C). PCA and t-SNE analyses exhibited recognizable distribution between the two groups (Figures 4D,E). In addition, patients in the low-risk group had superior OS than those with high-risk scores (Figure 4F). Moreover, the risk model demonstrated a well-predictive capability with area under curve (AUCs) of 0.81, 0.79, and 0.86 at 1, 3, and 5 years, respectively (Figure 4G).

We used the GSE10358 dataset to validate the capability of the 6-gene risk model. Patients were divided into high-risk (*n* = 44) and low-risk (*n* = 47) groups (Supplementary Figures 2A,B). In addition, the expression levels of *HNMT*, *TFEB*, *SDSL*, *SLC43A2*, and *SFXN3* were increased, while *TRH* expression was lower in the high-risk group (Supplementary Figure 2C). Similarly, the PCA and t-SNE analyses distributed patients in the two groups of the GSE10358 cohort in various directions (Supplementary Figures 2D,E). Patients in the low-risk group had a well-predictive value (*p* = 0.02) in the validation database (Supplementary Figure 2F). Additionally, the AUCs were 0.68, 0.66, and 0.77 at 1, 3, and 5 years, respectively, suggesting that the risk model had a good predictive capability for AML patient survival (Supplementary Figure 2G).

### Differences in clinical characteristics between the risk groups

To explore the correlation between the risk score and clinical characteristics, we assessed the differences in characteristics between the two risk subgroups. Patients in the high-risk group had older age, higher WBC counts, more patients who died, a higher percentage of M4, M5, and M6, advanced cytogenetic risk, and higher frequencies of NPM1 mutations (Table 2).

**TABLE 2:** The characteristics of 132 acute myeloid leukemia (AML) patients between different risk group.

Characteristics	High risk (*N* = 66)	Low risk (*N* = 66)	Total (*N* = 132)	*P*-value
Age	56.89 ± 15.71	49.65 ± 16.25	53.27 ± 16.33	0.01
WBC	39.94 ± 39.50	28.74 ± 44.58	34.38 ± 42.31	0.01
Blast cell (%)	68.50 ± 22.10	62.83 ± 24.06	65.67 ± 23.19	0.2
BM blast cell (%)	35.55 ± 29.79	40.68 ± 32.75	38.11 ± 31.29	0.44
Status				1.80E-04
Alive	15 (11.36%)	37 (28.03%)	52 (39.39%)	
Dead	51 (38.64%)	29 (21.97%)	80 (60.61%)	
FAB				1.60E-05
M0	4 (3.03%)	8 (6.06%)	12 (9.09%)	
M1	16 (12.12%)	16 (12.12%)	32 (24.24%)	
M2	10 (7.58%)	22 (16.67%)	32 (24.24%)	
M3	2 (1.52%)	12 (9.09%)	14 (10.61%)	
M4	20 (15.15%)	7 (5.30%)	27 (20.45%)	
M5	12 (9.09%)	0 (0.0e + 0%)	12 (9.09%)	
M6	2 (1.52%)	0 (0.0e + 0%)	2 (1.52%)	
M7	0 (0.0e + 0%)	1 (0.76%)	1 (0.76%)	
Cytogenetics risk				2.30E-05
Favorable	4 (3.03%)	26 (19.70%)	30 (22.73%)	
Intermediate	46 (34.85%)	27 (20.45%)	73 (55.30%)	
Poor	16 (12.12%)	13 (9.85%)	29 (21.97%)	
FLT3				0.09
Negative	41 (31.78%)	50 (38.76%)	91 (70.54%)	
Positive	24 (18.60%)	14 (10.85%)	38 (29.46%)	
NPMc				5.50E-04
Negative	41 (31.06%)	59 (44.70%)	100 (75.76%)	
Positive	25 (18.94%)	7 (5.30%)	32 (24.24%)	

**FIGURE 5:**
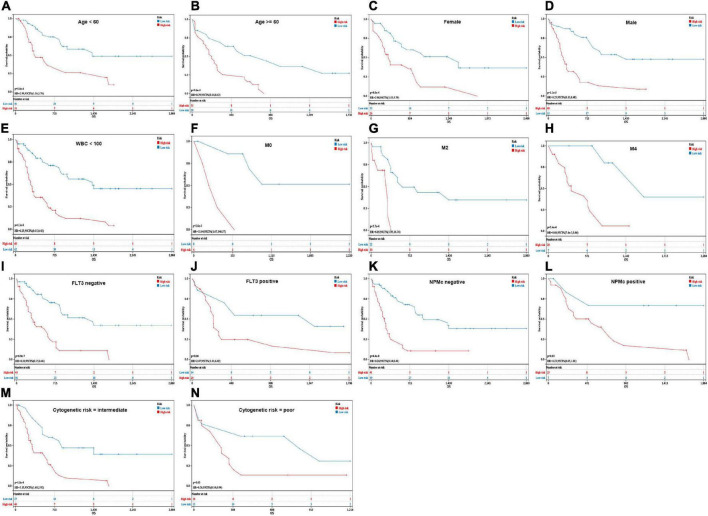
Kaplan–Meier analysis of OS in different groups. **(A,B)** Age (<60 years and ≥60 years). **(C,D)** Sex (female and male). **(E)** WBC counts (<100 × 10^9^/L). **(F–H)** French-American-British (FAB) classification (M0, M2, and M4). **(I,J)** FLT3 mutation (positive and negative). **(K,L)** NPMc mutation (positive and negative). **(M,N)** Cytogenetic risk (intermediate and poor).

Additionally, low-risk patients also had a better prognosis than high-risk patients and were still statistically significant in the following subgroups: age (<60 years: *p* = 5.8e-4; ≥60 years: *p* = 9.6e-4), sex (male: *p* = 8.0e-4; female: *p* = 1.3e-5), WBC counts (<100 × 10^9^/L: *p* = 1.3e-8), French-American-British (FAB) classification (M0: *p* = 2.0e-3; M2: *p* = 5.7e-4; M4: *p* = 5.4e-4), FLT3 mutation (positive: *p* = 0.04; negative: *p* = 6.9e-7), NPM1 mutation (positive: *p* = 0.03; negative: *p* = 4.4e-8), and cytogenetic risk (intermediate: *p* = 1.8e-4; poor: *p* = 0.03) (Figures 5A–N).

### Construction of a nomogram to predict survival

**FIGURE 6:**
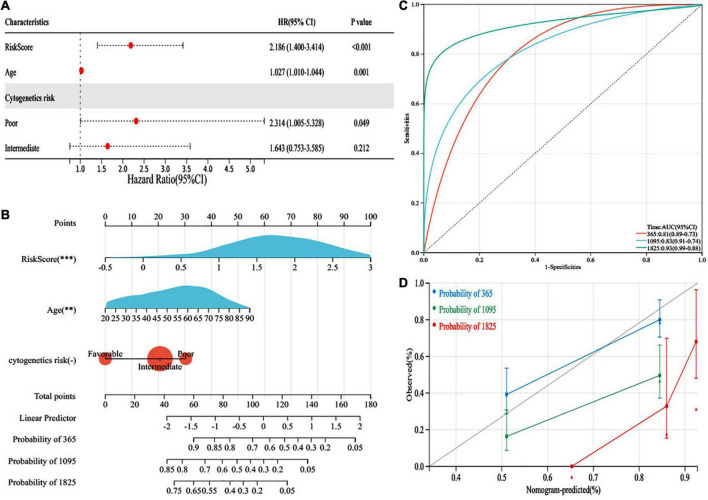
Development of a predictive nomogram in the cancer genome atlas (TCGA) datasets. **(A)** The multivariate cox regression analysis validated independent prognostic factors in TCGA database. **(B)** Development of nomogram based on risk score, age, and cytogenetic risk in TCGA database. **(C)** The performance of the nomogram was assessed by receiver operating characteristic (ROC) curves for 1, 3, and 5 years. **(D)** Calibration curves for 1-, 3-, and 5-year prediction. * *p* < 0.05, ^**^ *p* < 0.01, ^***^ *p* < 0.001, ns, no significant.

We performed a multivariate Cox regression analysis with the following variables: risk score, age, FAB, WBC counts, blast cell (%), cytogenetic risk, FLT3 mutation, and NPMc mutation. The risk score, age, and cytogenetic risk were independent impact factors for OS (Figure 6A). Furthermore, the risk model, age, and cytogenetic risk were integrated by a nomogram to predict the 1-, 3-, and 5-year OS rates of the AML patients more precisely (Figure 6B). Additionally, the nomogram displayed favorable prognostic capability, with AUCs of 0.81, 0.83, and 0.93 at 1, 3, and 5 years, respectively (Figure 6C). The calibration plot demonstrated that the nomogram was consistent with an ideal model (Figure 6D).

### Functions and pathways of the risk model

**FIGURE 7:**
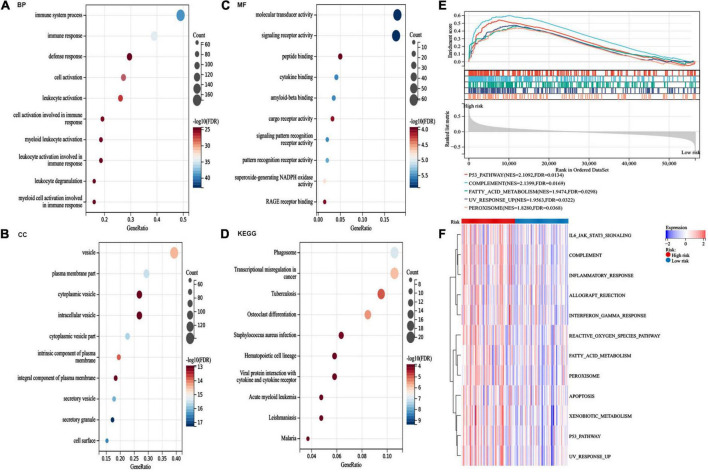
Pathway enrichment analysis between risk groups. **(A–C)** gene ontology (GO) analysis for differentially expressed genes (DEGs) between the two risk groups. **(D)** kyoto encyclopedia of genes and genomes (KEGG) pathway analysis for DEGs between the two risk groups. **(E)** The top five activated hallmark pathways by gene set enrichment analysis (GSEA). **(F)** Heatmaps of hallmark analysis in the risk groups by gene set variation analysis (GSVA) enrichment analyses.

The DEGs between the high-risk and low-risk groups were assessed to perform GO and KEGG analyses to explore the potential functions and pathways of the AA metabolism-related gene risk model. In addition, GSEA and GSVA of hallmark pathways were used to investigate the potential mechanisms. The DEGs of the risk subgroup participated in biological processes, such as immune system processes, immune responses, and defense responses, were mainly located in vesicles, plasma membrane parts, and granules and played roles in molecular transducer activity, signaling receptor activity, peptide binding, cytokine binding, and so on (Figures 7A–C). KEGG analysis revealed enrichment of phagosomes, transcriptional misregulation in cancer, osteoclast differentiation, hematopoietic cell lineage, and viral protein interactions with cytokines, and cytokine receptors (Figure 7D). GSEA found that the P53 pathway, complement, fatty acid metabolism, and inflammatory response were top enriched in the high-risk group (Figure 7E). Similarly, signaling pathways such as xenobiotic metabolism, reactive oxygen species pathway, fatty acid metabolism, peroxisome, JAK/STAT3, and inflammatory response were upregulated in the high-risk group (Figure 7F). These outcomes indicated that the AA metabolism-related 6-gene risk model was significantly concerned with cancer and the immunoregulation of the TME.

### Tumor immune microenvironment analysis of the risk model

**FIGURE 8:**
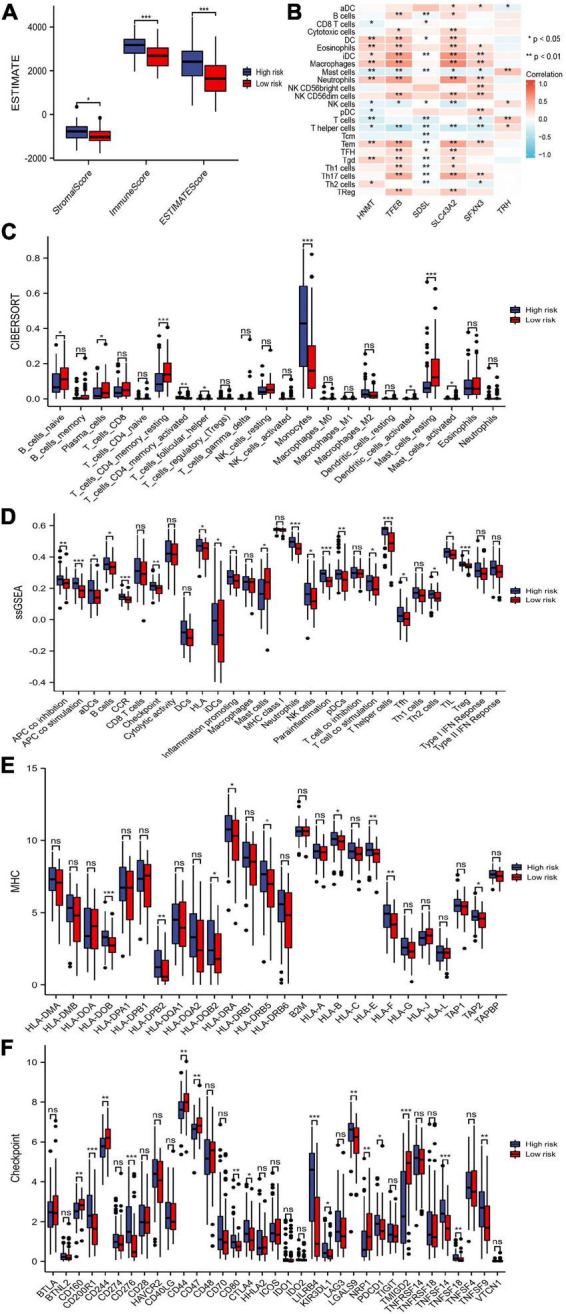
Immune cells and immune-related genes between risk groups. **(A)** Stromal score, immune score, and ESTIMATE score between the risk groups. **(B)** The abundance of immune cells between the risk groups. **(C,D)** Immune cells and immune responses between the risk groups by the CIBERSORT and single-sample gene set enrichment analysis (ssGSEA) algorithms. **(E,F)** Boxplots of HLA-related genes and checkpoint-related genes in the risk groups. * *p* < 0.05, ** *p* < 0.01, *** *p* < 0.001, ns, no significant.

We compared differences in the infiltrating immune cells and immune-related functions in the two groups. AML patients in the high-risk group had significantly higher stromal, immune, and ESTIMATE scores according to the ESTIMATE algorithm (Figure 8A). The association between the six genes in the risk model and the abundance of immune cells was also evaluated, and partial immune cells were found to be related to the six genes (Figure 8B). Moreover, the CIBERSORT algorithm found that the high-risk group had a more abundance of infiltrating immune cells in monocytes and active CD4+ memory T cells but inferior infiltration of naïve B cells, plasma cells, resting memory CD4+ T cells, follicular helper T cells, activated mast cells, and resting mast cells (Figure 8C). Additionally, the ssGSEA algorithm suggested that tumor-infiltrating cells, Tregs, checkpoint, inflammation-promoting, APC, HLA, and IFN responses were highly active in the high-risk group (Figure 8D). These results revealed that the high-risk group had higher immune infiltration and can be recognized as an immune “hot” phenotype.

The expression of HLA-related genes and immune checkpoint-related genes between two subgroups were also evaluated. We showed that most HLA-related genes were upregulated in the high-risk group (Figure 8E). In addition, patients in the high-risk group possessed significantly higher expression of *PDCD1* (*PD1*), *CTLA4*, *CD200R1*, *CD276*, *CD80*, *LILRB4*, *KIR3DL1*, *LGALS9*, *TNFSF14*, *TNFSF18*, and *TNFSF9*. The expression levels of *CD160*, *CD244*, *CD44*, *CD47*, *NRP1*, and *TMIGD2* was lower in high-risk patients than in low-risk patients (Figure 8F).

### Tumor mutation burden and drug sensitivity analyses of the risk model

**FIGURE 9:**
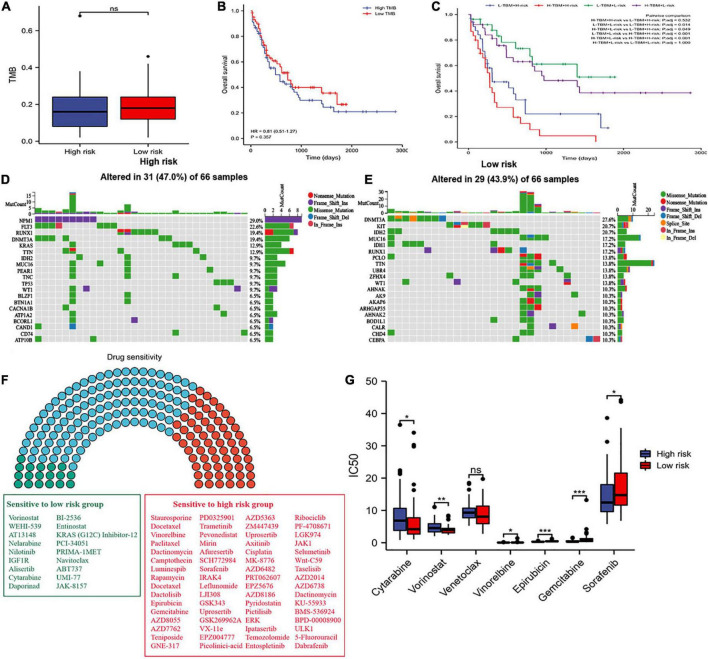
Tumor mutation burden (TMB) and drug sensitivity analysis. **(A)** Difference in TBM between the two risk groups. **(B)** OS of patients with different TMB levels. **(C)** Kaplan–Meier analysis of OS with TMB and risk score. **(D,E)** Waterfall plot of the top twenty genes’ TMB status in the two risk groups. **(F)** Drug sensitivity analyses between the two risk groups. Green, sensitive drugs in low-risk group; Red, sensitive drugs high-risk group; Blue, no sense. **(G)** Comparison of drug sensitivity between the two risk groups. * *p* < 0.05, ** *p* < 0.01, *** *p* < 0.001, ns, no significant.

We did not find significantly different between the two risk groups in the level of TMB (Figure 9A). The patients’ survival of different TMB levels was no significant difference (Figure 9B). According to risk scores and TMB scores, patients were divided into four groups. We exhibited that the low-mutation-low-risk group had the highest survival, while the high-mutation-high-risk group had the lowest survival (Figure 9C). The results indicated that our model’s prediction ability was more substantial than the TBM’s. We explored the different distribution of somatic mutations in the two risk groups. Patients in the high-risk group showed more somatic mutations in *NPM1*, *FLT3*, *KRAS*, and *TP53* than those in the low-risk group and lower mutations in *DNMT3A* (Figures 9D,E).

Additionally, we propagated predictions from GDSC2 data into TCGA samples, and the IC50 values of 198 chemotherapy drugs were calculated (Figure 9F). We suggested that 64 drugs had lower IC50 values in the high-risk group, and 18 drugs in the low-risk group. Moreover, we selected commonly used medications in AML to evaluate the sensitivity of these drugs. We discovered that patients in the low-risk group had significantly lower IC50 values of cytarabine and vorinostat than those in the high-risk group, and had higher IC50 values of vinorelbine, epirubicin, gemcitabine, and sorafenib in the low-risk group (Figure 9G). We also found that patients in the low-risk group had lower IC50 values of venetoclax, but without statistical significance, which consistent with the result where the expression of BCL2 was higher in the low-risk group than in the high-risk group (Supplementary Figure 3) and the apoptosis was more active in the low-risk group than in the high-risk group (Figure 7F). These results offer evidence for the treatment stratification of patients with AML.

### Distribution of amino acid pathways between the two risk groups

**FIGURE 10:**
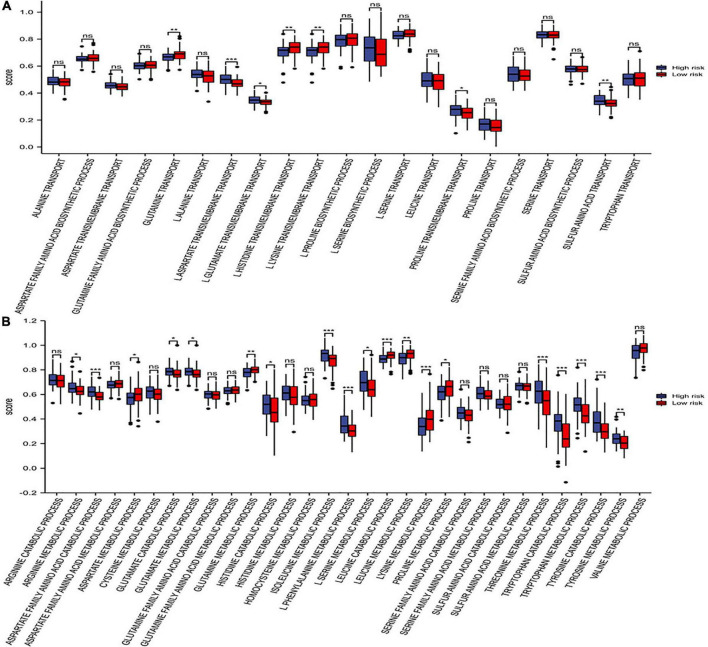
Distribution of amino acid pathways. **(A)** The single-sample gene set enrichment analysis (ssGSEA) analysis of the amino acid synthetic and transport pathways. **(B)** The ssGSEA analysis of the amino acid catabolic and metabolic pathways. * *p* < 0.05, ** *p* < 0.01, *** *p* < 0.001, ns, no significant.

The differences in amino acid pathway activity between the two risk subgroups were exhibited based on ssGSEA. We demonstrated that the processes of glutamine transport, L-lysine transmembrane transport, L-histidine transmembrane transport, and proline transmembrane transport were more active in the patients with low-risk scores. In contrast, the processes of L-aspartate transmembrane transport, sulfur amino acid transport and L-glutamate transmembrane transport were more active in the high-risk group (Figure 10A). Furthermore, the aspartate family amino acid catabolic, arginine metabolic, glutamate catabolic, glutamate metabolic, histidine catabolic, isoleucine metabolic, L-phenylalanine metabolic, L-serine metabolic, threonine metabolic, tryptophan catabolic and metabolic, tyrosine catabolic and metabolic processes were more active in the high-risk subgroup. In contrast, the aspartate metabolic, glutamine metabolic, leucine catabolic, leucine metabolic, lysine metabolic and proline metabolic processes were more active in the low-risk subgroup (Figure 10B). In conclusion, some amino acid metabolism pathways and risk scores were positively associated with the malignant progression of AML.

## Discussion

Although targeting cancer metabolism to increase immunotherapy efficacy and overcome drug resistance is highly promising, targeting cancer metabolism may disrupt normal metabolic pathways in immune cells in the TME (30). Therefore, targeting the appropriate metabolic pathways and molecules to kill tumor cells without damaging antitumor immunity is critical. Studies have reported that the concentration of all 20 amino acids is higher in the BM than in peripheral blood, and plays a positive role in the proliferation and maintenance of hemopoietic stem cells (HSCs) (31). In addition, leukemia stem cells (LSCs) in newly diagnosed AML patients, are dependent on amino acid metabolism for OXPHOS to survival (15). Moreover, therapies targeting several amino acid metabolisms can kill AML blast and LSCs, including the glutaminase (GLS) inhibitor CB-839 (32). Glutaminase inhibition can promote leukemia cells to overcome drug resistance, and to be sensitive to BCL2 inhibitors, FMS-like tyrosine kinase 3 and other tyrosine kinase inhibitors (25, 32, 33). Moreover, tryptophan metabolic enzyme and IDO inhibitor potentiate the effects of CD33/CD3-bispecific T-cell engage (BiTE^®^) antibodies (34). IDO1 inhibitors combined with CD19 CAR-T immunotherapy improved the efficacy of CD19 CAR-T–cell therapy in mouse lymphoma xenograft models (35). The polyethylene glycol–conjugated (PEGylated) forms of arginine deiminase (ADI) (ADI-PEG 20) combined with cytarabine to treat AML (25), and PEG-ARGase I (arginase-1) (36). These studies indicated that AA metabolism was an ideal target to improve cancer immunotherapy, but there are still few related multi-omics comprehensive studies about AML and even other tumors.

We first identified two clusters based on prognosis-related genes of AA metabolism. The two clusters showed significantly different clinical characteristics, AA metabolism, TME, and pathways. The immune phenotype changed from “hot” to “cold” from clusters C1 to C2, accompanied by low expression of most AA metabolism-related genes and inhibition of tumor signaling pathways. In addition, the “hot” immune phenotype in cluster C1 is usually correlated with poor survival, older age, and poor cytogenetic risk. Increasing evidence indicates that heterogeneity in the immune environment and its interaction with AML blasts cause different outcomes and responses to therapy. Our results found that cluster C1 possessed more monocytes, Tregs, and neutrophils, which are related to immune suppression. However, cluster C1 possessed fewer naïve B cells, plasma cells, resting memory CD4+ T cells, resting NK cells, activated mast cells, and resting mast cells, which are acknowledged as antitumor immune cells (21). Immunologically hot or cold tumor microenvironments may have various prognostic effects for checkpoint inhibitor (CPI) therapy in solid tumors. However, the heterogeneity of the AML microenvironment was little known (37). AML BM samples had two types of immune microenvironments, one type was an immune-enriched and IFN-γ dominant type, which had elevated expression of lymphocyte-associated genes, IFN-γ, and immune checkpoint molecules; the other type was an immune-depleted type, which had low expression of T-cell and B-cell genes, while had elevated expression of mast cell function– and T-cell exhaustion–associated genes (38, 39). Similarly, we demonstrated that checkpoint-related and tumor-infiltrating lymphocyte-associated genes were highly active in cluster C1, suggesting that patients in cluster C1 were immune-enriched and might be more susceptible to CPIs.

We filtered six essential genes to develop the risk model *via* LASSO analyses based on the results of univariate Cox analysis and DEGs, including *TRH*, *HNMT*, *TFEB*, *SDSL*, *SLC43A2*, and *SFXN3*. Based on the risk model, we calculated risk scores and divided patients into low- and high-risk groups. The patients with high-risk scores had a poor prognosis, older age, poor cytogenetic risk, and high AUC. We displayed that the risk model was an independent prognostic factor for AML by multivariate Cox analysis and clinical characteristics and even in the nomogram, which integrated the risk score with clinical characteristics. These results revealed that our model was firm and accomplished favorable in predicting the survival of AML patients.

The functions of six genes in the model and their related proteins were explored. *Thyrotropin Releasing Hormone* (*TRH*) encodes a member of the thyrotropin-releasing hormone family. A study showed that high *TRH* expression was related to favorable survival in t (8; 21) (q22; q22) AML (40), which was consistent with our results. *HNMT* (*Histamine N-Methyltransferase*) encodes histamine N-methyltransferase, which is found in the cytosol and uses S-adenosyl-L-methionine as the methyl donor and participates in histamine metabolism. *HNMT* significantly upregulated in human non-small cell lung cancer (NSCLC) tissues, conferred a worse prognosis, and was co-expressed with *HER2* (41). In addition, *HNMT* upregulation causes cancer stem cell formation and protect it from oxidative stress by interaction with *HER2* in NSCLC (41). *TFEB* (*Transcription Factor EB*) enables DNA-binding transcription factor activity. A study reported that homocysteine suppresses autophagy through *AMPK*-*mTOR*-*TFEB* signaling in human THP-1 macrophages (42). Moreover, disrupting the *MYC*-*TFEB* circuit affects amino acid homeostasis and provokes metabolic energy (43). *SDSL* (*Serine Dehydratase Like*) is predicted to be involved in isoleucine biosynthetic and threonine catabolic processes. *SLC43A2* (*Solute Carrier Family 43 Member 2*) encodes a member of the L-amino acid transporter-3 or SLC43 family of transporters. It mediates transport of L-isomers of neutral amino acids, including leucine, phenylalanine, valine, and methionine, and don’t depend on sodium, chloride, and pH (44). Cancer *SLC43A2* can alter T-cell methionine metabolism and histone methylation (45). *SFXN3* (*Sideroflexin 3*) enables serine transmembrane transporter activity and is involved in serine import into mitochondria. Serum *SFXN3* autoantibody is a novel tumor marker for oral squamous cell carcinoma (46). The function of *TRH* in AML has been verified by previous studies. However, there are no direct reports of *HNMT*, *TFEB*, *SDSL*, *SLC43A2*, and *SFXN3* in AML, and this study was the first to report that *HNMT*, *TFEB*, *SDSL*, *SLC43A2*, and *SFXN3* were related to the prognosis of AML. Our results might define new biomarkers of AML to be explored for further research.

GO and KEGG enrichment analyses suggested that the genes in the high-risk group mainly participated in immune system processes and played roles in molecular transducer activity and signaling receptor activity. Combining the results of GSVA and GSEA of hallmark pathways, the xenobiotic metabolism, reactive oxygen species (ROS) pathway, fatty acid metabolism, JAK/STAT3, and inflammatory response were upregulated in the high-risk group. Study showed that polymorphic variants in xenobiotic metabolism genes may increase the risk of adult AML (47). Cancer cells need high ROS levels to promote tumor development and progression (48). AML is usually under oxidative stress because of impaired ROS homeostasis (49, 50). BM adipocytes were often been observed to remodeling and lipolysis in elderly AML patients, and AML cell survival through metabolic activation of fatty acid oxidation (FAO). This can cause dormancy and drug resistance in LSCs (51). The JAK/STAT pathway is abnormally activated or suppressed in LSCs, and plays a vital role in AML survival, proliferation, and self-renewal properties (52). The inflammatory population is associated with poor prognosis in AML patients (53). These results indicated the interference of nutrient metabolism of patients in the high-risk group, and proliferation and survival activity of tumor cells were more active in the high-risk group. These pathways might be critical antitumor ways for targeting amino acid metabolic therapy.

Similarly, the high-risk group was recognized as having a “hot” immunophenotype related to poor survival, while the low-risk group exhibited the opposite effect. Our results found that the high-risk group possessed more monocytes, Tregs. and neutrophils and higher expression of PD1 and CTLA4, which were related to immune suppression (37). The low-risk group exhibited infiltration of naïve B cells, plasma cells, resting memory CD4+ T cells, activated mast cells, and resting mast cells, which are acknowledged as antitumor immune cells (21). Meanwhile, checkpoint-related and HLA-related genes were also highly active in the high-risk group, which indicated that more recognition of tumor-associated antigens with more HLA presentation, and more success of immune checkpoint inhibitor therapy (54). Furthermore, TMB is the total load of somatic mutations in tumor cells, which may cause specific tumor neoantigens. Therefore, patients with a high TMB are likely to be more responsive to immunotherapy (54–57). Our results observed that the risk score was not associated with TBM, suggesting that the predictive ability of the risk model is independent of TMB and that the risk score can replenish the potential patients. They can benefit from the current conditions. Additionally, the NPM1 gene is the most common genetic lesion in adult AML, occurring in approximately one-third of patients (58), and NPM1 mutation leads to an abnormal cytoplasmic expression that leads to more efficient HLA presentation (59). Our results demonstrated that NPM1 was the most mutated in the high-risk group and that HLA-related genes were highly active in the high-risk group. Thus, patients in the high-risk group might benefit more from immunotherapy. The results of drug sensitivity prediction demonstrated that the patients in the high-risk group were more sensitive to sorafenib, selumetinib, and entospletinib. Sorafenib, a multitargeted FLT3 kinase inhibitor, either alone or in combination with conventional chemotherapy, plays a crucial role in AML therapy (60, 61). Selumetinib is an oral MAP-ERK kinase (MEK)-1/2 inhibitor and it has modest antileukemic activity in advanced AML in a phase II study (62). Entospletinib was an inhibitor of SYK, and AML patients with higher *HOXA9/MEIS1* expression had improved overall survival than lower *HOXA9/MEIS1* expression, when patients received entospletinib in combination with intensive chemotherapy in a phase Ib/II study (63). The correlation between amino acid metabolism and the efficacy of sorafenib, selumitinib and entospletinib requires further investigation. We also found that patients in the high-risk group had higher IC50 values of venetoclax (the BCL-2 inhibitor), which consistent with the result where the expression of *BCL2* was higher in the low-risk group than in the high-risk group and the apoptosis was more active in the low-risk group than in the high-risk group. These results suggested that although in the presence of apoptosis, AML cells could dependent on amino acid metabolism to survival. However, the potential mechanism of this phenomenon needed to further explored.

Abnormal AA metabolism can induce immune escape and drug resistance (64, 65). First, glutaminase is upregulated expression in AML (25), and glutamine metabolism is important for the maintenance, relapse, and refractory of leukemia (15, 32, 66, 67). Furthermore, glutamine is also essential for immune cells, such as T cells and macrophages (68, 69). Glutamine restriction disturbs the balance between the generation of Th1 cells and Treg cells, and promotes producing more Treg phenotype (70, 71). Glutamate is a glutamine-derived substance, and we demonstrated that glutamate metabolism was active in the high-risk group. Targeting glutamine metabolism may be effective in leukemia (24). Furthermore, metabolic adaptation can cause several tyrosine kinase inhibitors (TKIs) resistance, so targeting glutaminolysis combined with TKIs in specific leukemias can be effective (25). Second, AML blasts depend on arginine for proliferation (36), and they have deficiencies in arginine-recycling pathway enzymes, which means that they are arginine auxotrophic (36). In addition, activated T cells increase the metabolic requirement for arginine (72, 73). In our results, arginine metabolism was active in the high-risk group. ADI and ARGase are two crucial enzymes of the arginine metabolism/urea cycle, could be targeted for potential therapy, such as ADI-PEG 20 (25) and PEG-ARGase I (36). Third, indoleamine-2,3-dioxygenase (IDO) 1, IDO2, and tryptophan-2,3-dioxygenase are the limiting enzymes for tryptophan metabolism (74). AML patients express IDO, which is related to significantly poor survival (34). AML cells and leukemic DCs directly convert T cells into Treg cells and inhibit T-cell proliferation by an IDO-dependent mechanism (75, 76). The mesenchymal stromal cells (MSCs) upregulated IDO to suppress T-cell function when response to inflammation (77). Our results showed that tryptophan metabolism was active in the high-risk group. Moreover, an IDO inhibitor enhances the effects of CD33/CD3-bispecific T-cell engage (BiTE^®^) antibodies (34). Additionally, cysteine metabolism is vital for LSC maintenance, and consumption of cysteine canuses the death of AML stem and progenitor cells (78). AAs are necessary for the development of hematological malignancies by supplying energy, promoting biosynthesis, and assisting immune escape. Hematological malignancies usually depend on specific amino acids for their survival, and targeting AA metabolism may be a promising option that has been clinically validated (15).

Undeniably, there were some limitations in our study. First, we downloaded data from public databases. The AA metabolism-related gene risk model and its association with immunotherapy response need to be explored and validated by more experimental and clinical studies. Second, the results of amino acid metabolism in AML patients need to be verified. More practical and clinical studies should be performed to explore further the potential effect of amino acid metabolism on the prognosis and immune therapy response of AML.

In conclusion, this article finally developed an AA metabolism-related risk signature to predict the prognosis of patients with AML and explored its essential role in TME. Our results could offer novel perspectives for individual precision medical options. Meanwhile, the risk model can supplement identifying potential patients who can benefit from immunotherapy. Furthermore, related targeted drugs could be explored according to new tumor-associated biomarkers and changes in amino acid metabolic pathways, and based on different amino acid metabolism in AML patients, and individual precision medical options could be carried out.

## Data availability statement

The datasets presented in this study can be found in online repositories. The names of the repository/repositories and accession number(s) can be found in the article/Supplementary material.

## Author contributions

HZ and TN participated in the study design. HZ wrote the manuscript. HZ and FW acquired, analyzed, and interpreted the data. TN revised the manuscript. All authors contributed to the manuscript and approved the submitted version.

## Footnotes

1
https://portal.gdc.cancer.gov/

2
http://www.gsea-msigdb.org/gsea/msigdb/index.jsp

3
https://www.kegg.jp/kegg/rest/keggapi.html

4
http://software.broadinstitute.org/gsea/index.jsp

5
https://www.cancerrxgene.org/
