# Supplementary material for: Prediction of prognosis and immunotherapy response of amino acid metabolism genes in acute myeloid leukemia
Source: Front Nutr. 2022 Dec 22;9:1056648. doi: 10.3389/fnut.2022.1056648 (PMC9815546; doi:10.3389/fnut.2022.1056648)
Supplement: Supplementary file 1 [file Data_Sheet_1.docx]

Supplementary Material


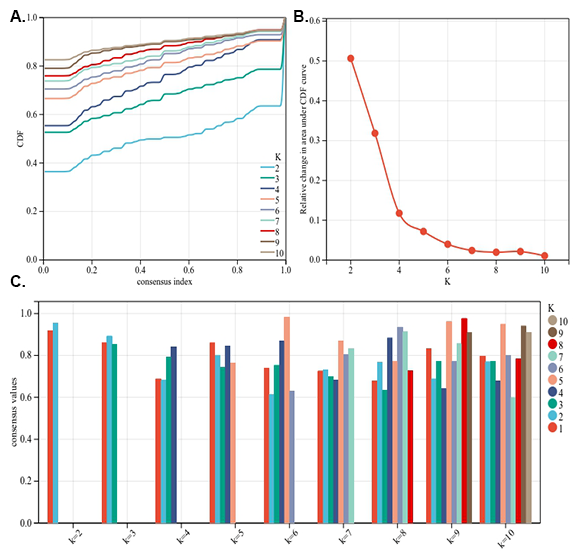


**Supplementary Figure 1.** the consensus CDF curve (A), delta area (B), and optimal number in Nbclust (C).


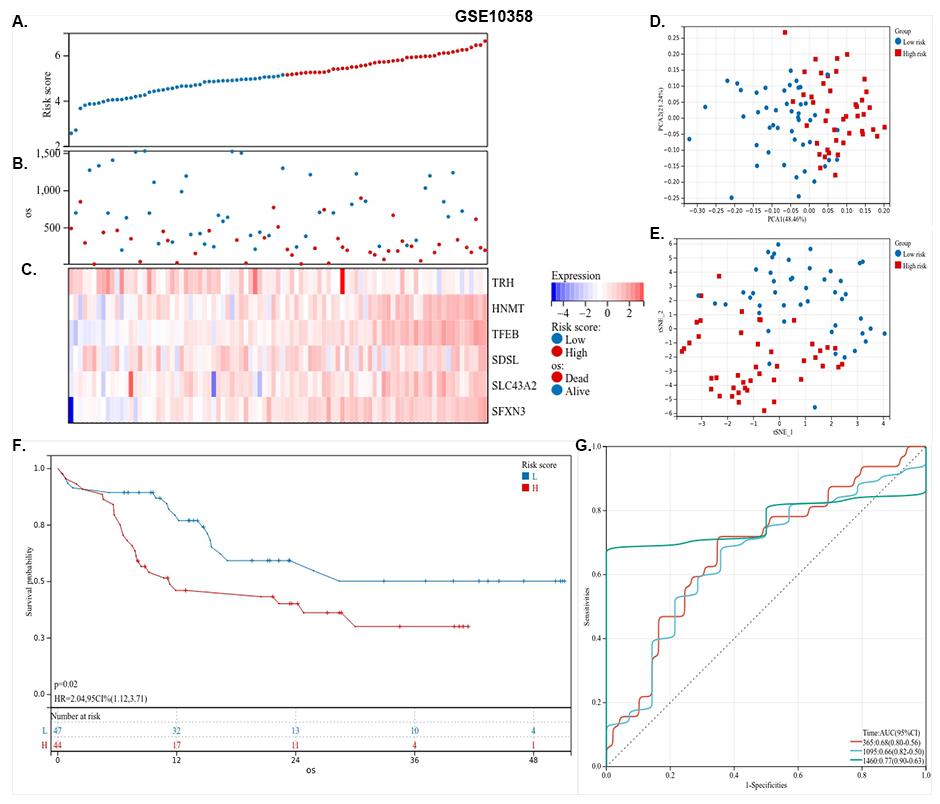


**Supplementary Figure 2. Prognostic study of GSE10358 dataset via the 6-gene risk model. (A)** Risk scores, **(B)** survival status and **(C)** Heatmap of the 6 survival-related amino acid metabolism DEGs. **(D)** PCA and **(E)** t-SNE analysis showing the different gene expression of samples. **(F)** Kaplan–Meier OS curves for high-risk and low-risk groups. **(G)** The 1-, 3-, and 5-year ROC curve to predict the survival status.


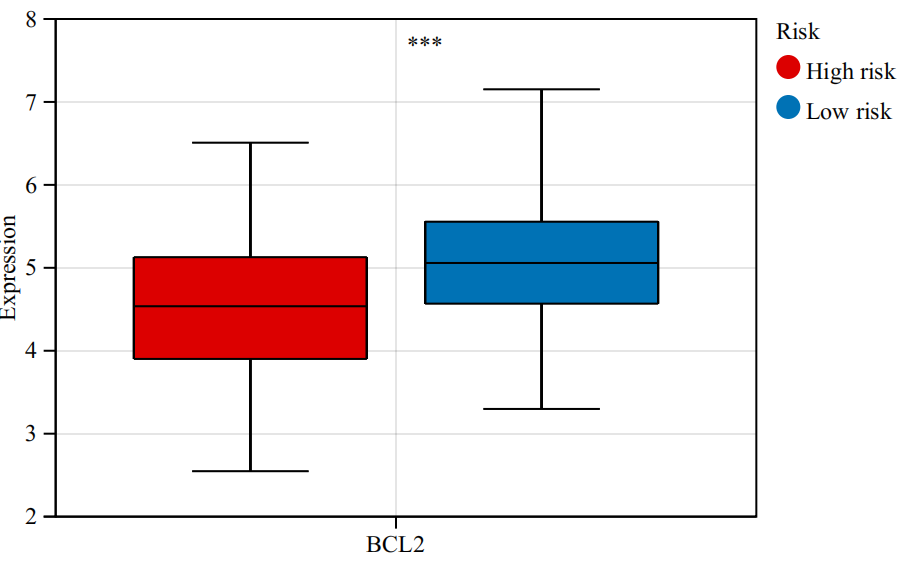


**Supplementary Figure 3. The expression of BCL-2 between the two risk groups.**

**Supplementary Table 1. Amino acid metabolism-related genes.**

| Pathways | Genes |
| --- | --- |
| GOBP_AMINO_ACID_ACTIVATION | FARSB, WARS2, FARS2, TARS3, EARS2, AASDH, AARS1, DARS1, EPRS1, FARSA, LARS2, HARS2, PARS2, GARS1, GATC, HARS1, IARS1, KARS1, MARS1, NARS1, YARS2, LARS1, GATB, SARS2, DALRD3, DARS2, QRSL1, IARS2, RARS2, VARS2, LRRC47, AARS2, QARS1, RARS1, SARS1, TARS1, VARS1, WARS1, CARS2, NARS2, TARS2, AARSD1, CARS1, YARS1, MARS2 |
| GOBP_AMINO_ACID_HOMEOSTASIS | KCTD7, SLC7A11, GLS, GRM2, SLC66A1, SLC1A1, TPP2 |
| GOBP_AMINO_ACID_IMPORT | ARL6IP5, SLC6A14, SFXN2, SLC36A4, SLC43A2, AGT, CLN8, SLC36A1, TSPO2, ARL6IP1, SLC7A8, SLC7A11, SH3BP4, GFAP, GRM1, ITGB1, KCNJ10, ATP1A2, NTSR1, SLC6A20, SLC25A38, SLC47A1, PSEN1, CLTRN, ACE2, RGS2, RGS4, SLC1A1, SLC1A2, SLC1A3, SLC1A4, SLC1A5, SLC1A6, SLC3A2, SLC6A1, SLC6A6, SLC6A9, SLC6A11, SLC6A12, SLC6A13, SLC7A1, SLC7A2, SLC16A2, SLC22A2, SLC22A4, TNF, SLC7A5, SFXN3, SLC7A3, SLC43A1, PER2, SLC6A5, SLC9A3R1, SFXN1 |
| GOBP_AMINO_ACID_IMPORT_ACROSS_PLASMA_MEMBRANE | ARL6IP5, SLC6A14, SLC36A4, SLC43A2, AGT, CLN8, SLC36A1, TSPO2, ARL6IP1, SLC7A8, SLC7A11, GFAP, GRM1, ITGB1, KCNJ10, ATP1A2, NTSR1, SLC6A20, SLC47A1, PSEN1, CLTRN, ACE2, RGS2, RGS4, SLC1A1, SLC1A2, SLC1A3, SLC1A4, SLC1A5, SLC1A6, SLC3A2, SLC6A6, SLC6A9, SLC6A13, SLC7A1, SLC7A2, SLC16A2, SLC22A2, SLC22A4, TNF, SLC7A5, SLC7A3, SLC43A1, PER2, SLC6A5 |
| GOBP_AMINO_ACID_SALVAGE | BHMT2, MTAP, APIP, ADI1, ENOPH1, BHMT, MRI1 |
| GOBP_AMINO_ACID_TRANSMEMBRANE_TRANSPORT | SLC25A13, SLC25A15, ARL6IP5, SLC38A3, SLC7A9, PRAF2, SLC6A14, SLC16A10, SFXN2, CLN3, SLC36A4, SLC15A4, SLC25A29, SLC38A10, SLC43A2, MFSD12, SLC32A1, SLC38A6, SLC38A8, SLC38A11, SLC66A1L, SLC38A9, SLC36A2, SLC7A13, AGT, CLN8, SLC36A1, TSPO2, ARL6IP1, SLC7A8, SLC7A11, SLC17A8, GFAP, SLC36A3, GRM1, SLC6A19, SLC6A18, ITGB1, KCNJ10, SLC7A5P2, ATP1A2, NTSR1, SLC38A2, SLC6A20, SLC66A1, SLC25A38, SLC38A4, SLC6A15, LRRC8D, SLC38A7, SLC47A1, LRRC8A, SLC7A10, PSEN1, SLC17A7, SLC17A6, CLTRN, SLC7A14, ACE2, RGS2, RGS4, SLC1A1, SLC1A2, SLC1A3, SLC1A4, SLC1A5, SLC1A6, SLC1A7, SLC3A1, SLC3A2, SLC6A6, SLC6A7, SLC6A9, SLC6A12, SLC6A13, SLC7A1, SLC7A2, SLC7A4, SLC16A2, SLC22A2, SLC22A4, TNF, SLC25A22, LRRC8E, SLC7A5, SLC38A1, SFXN3, SLC7A5P1, SLC25A18, SLC25A2, LRRC8C, SLC7A3, SLC43A1, SLC25A12, PER2, SLC7A7, SLC7A6, SLC6A5, SLC38A5, SFXN1 |
| GOBP_AMINO_ACID_TRANSPORT | SLC25A13, SLC25A15, ARL6IP5, PDPN, GIPC1, SERINC3, SLC38A3, SLC7A9, PRAF2, SLC6A14, HRH3, SLC16A10, SFXN2, SFXN4, CLN3, SLC36A4, SLC15A4, SLC25A29, SLC38A10, SLC43A2, MFSD12, ADORA1, ADORA2A, SLC32A1, SLC38A6, SLC38A8, CTNS, SLC38A11, SLC66A1L, SLC38A9, SLC36A2, SLC25A48, SLC7A13, ABAT, AGT, CLN8, SLC36A1, TSPO2, RAB3GAP1, ARL6IP1, SLC7A8, SLC7A11, SH3BP4, SLC17A8, GABBR1, SERINC5, SLC17A5, GFAP, GJA1, SLC25A45, SLC25A47, SLC36A3, GRM1, GRM2, GRM7, APBA1, HTR1B, SLC6A19, SLC6A18, ITGB1, KCNJ10, SLC7A5P2, SLC6A17, LEP, LLGL2, MYC, NF1, ATP1A2, NPY5R, NTRK2, NTSR1, OCA2, P2RX7, SLC38A2, SLC6A20, SLC66A1, SLC25A38, SLC38A4, AVP, SLC6A15, LRRC8D, AVPR1A, SLC38A7, SLC47A1, AVPR1B, LRRC8A, SLC7A10, PSEN1, SLC17A7, SLC17A6, CLTRN, SLC7A14, ACE2, RGS2, RGS4, SLC1A1, SLC1A2, SLC1A3, SLC1A4, SLC1A5, SLC1A6, SLC1A7, SLC3A1, SLC3A2, SLC6A1, SLC6A6, SLC6A7, SLC6A9, SLC6A11, SLC6A12, SLC6A13, SLC7A1, SLC7A2, SLC7A4, SLC12A2, SLC16A2, SLC22A2, SLC22A4, SNCA, STXBP1, SYT4, TNF, TRH, TRPC4, TRPV1, XK, SLC25A22, LRRC8E, SLC7A5, SLC38A1, SFXN3, SLC7A5P1, SLC25A18, SLC25A2, DTNBP1, LRRC8C, SLC7A3, SLC43A1, KMO, SLC25A12""PER2, SLC7A7, SLC7A6, SLC6A5, SLC38A5, SLC9A3R1, SFXN1, SFXN5, SLC25A44, SV2A |
| GOBP_CELLULAR_AMINO_ACID_BIOSYNTHETIC_PROCESS | AASS, CBSL, SERINC3, SDS, ILVBL, PARK7, SDSL, CLN3, NOXRED1, CPS1, GOT1L1, CTH, NAGS, DHFR, ABAT, DPYD, AGXT, DHFR2, ALDH1A1, SEPHS2, SEPHS1, BHMT2, SERINC5, PHGDH, GGT1, GLS2, GLS, GLUD1, GLUD2, GLUL, GOT1, GOT2, PYCR2, PSAT1, ASL, ASNS, MTHFD2L, ASS1, MTAP, MTHFD1, MTHFR, MTR, MTRR, ATP2B4, OAT, OTC, PAH, PCBD1, APIP, LGSN, UPB1, PLOD2, ASNSD1, ADI1, PSPH, DHFRP1, PYCR1, ALDH18A1, ENOPH1, BCAT1, BCAT2, AASDHPPT, BHMT, SRR, SHMT1, SHMT2, AGXT2, SLC1A3, PYCR3, CAD, PCBD2, MRI1, SLC25A12, CBS, PSPHP1, PLOD3 |
| GOBP_CELLULAR_AMINO_ACID_CATABOLIC_PROCESS | AASS, RIDA, CBSL, BCKDK, CDO1, FTCD, SDS, HIBADH, HOGA1, SDSL, HYKK, AFMID, ACMSD, UROC1, ADHFE1, CARNMT1, CRYM, AMDHD1, TDH, DAO, DBT, IDO2, DLD, DLST, SARDH, ABAT, AGXT, ECHS1, ETFA, ETFB, FAH, GCAT, HAAO, DDAH2, DDAH1, GAD1, GAD2, IL4I1, HIBCH, GCSH, ACAD8, GLS2, GLDC, GLS, GLUD1, GLUD2, AMT, GLUL, GOT1, GOT2, GPT, GSTZ1, HSD17B10, HAL, HDC, HGD, HMGCL, HNMT, HPD, ACADSB, IDO1, IVD, ACAT1, ARG1, ARG2, MIR21, MAT1A, ALDH6A1, ASPA, MTRR, NOS1, NOS2, NOS3, ATP2B4, OAT, OTC, PAH, AADAT, PIPOX, CSAD, HMGCLL1, AUH, ENOSF1, PRODH, KYAT3, MCCC1, CARNS1, PRODH2, QDPR, BCAT2, BCKDHA, BCKDHB, MCCC2, BLMH, ALDH8A1, SHMT1, SHMT2, AGXT2, TAT, TDO2, ALDH5A1, ASRGL1, GPT2, DDO, KMO, ALDH4A1, CBS, KYAT1, KYNU, ARHGAP11B, SLC25A21, SLC25A44 |
| GOBP_CELLULAR_AMINO_ACID_METABOLIC_PROCESS | GLYATL1B, FARSB, AASS, SLC25A13, RIDA, GLYAT, CBSL, BCKDK, WARS2, CDO1, MTHFS, FARS2, FTCD, SERINC3, SDS, ILVBL, HIBADH, HOGA1, PARK7, AZIN2, SDSL, CLN3, TPH2, NOXRED1, TARS3, HYKK, EARS2, AFMID, ACMSD, UROC1, AASDH, CPS1, GOT1L1, ADHFE1, CARNMT1, CRYM, TTC36, AMDHD1, PM20D1, CTH, CTNS, CTPS1, TDH, ADSS2, AARS1, DAO, DARS1, NAGS, DBT, DCT, DDC, IDO2, DHFR, DIO1, DLD, DLST, SARDH, ABAT, DPEP1, DPYD, AGXT, ECHS1, DHFR2, EPRS1, ETFA, ETFB, ALDH1A1, FAH, FARSA, SEPHS2, SEPHS1, LARS2, SIRT4, HARS2, ICMT, GCAT, HAAO, FPGS, DDAH2, DDAH1, SLC7A11, BHMT2, SERINC5, GAD1, GAD2, IL4I1, PARS2, GARS1, GART, PHGDH, BLOC1S6, HIBCH, GCDH, GCSH, GFPT1, GGT1, GGT5, ACAD8, GLS2, GNMT, GCLC, GCLM, GLDC, GLS, GLUD1, GLUD2, AMT, GLUL, GOT1, GOT2, GATC, RIMKLA, GPT, GSS, GSTZ1, PYCR2, PSAT1, HSD17B10, HAL, HARS1, HDC, HGD, HMGCL, HNF4A, HNMT, HPD, IARS1, NAT8L, ACADSB, IDO1, INS, IVD, KARS1, ASPG, ACAT1, ARG1, ARG2, IYD, ACCSL, MIR21, MARS1, MAT1A, ART4, MECP2, ALDH6A1, ASL, MPST, ASNS, MTHFD2L, ASPA, ASS1, MSRA, MTAP, MTHFD1, MTHFR, MTR, MTRR, MMUT, NARS1, ATF4, NOS1, NOS2, NOS3, ATP2B4, OAT, ODC1, OTC, NOX4, PAH, PCBD1, YARS2, APIP, THAP4, AADAT, PIPOX, CSAD, LARS1, SCLY, LGSN, AZIN1, UPB1, PEPD, GATB, PFAS, PLOD2, ATP7A, HMGCLL1, ASNSD1, PPAT, AUH, SARS2, DALRD3, DARS2, ADI1, THNSL2, QRSL1, ENOSF1, IARS2, PRODH, KYAT3, CTPS2, MCCC1, NIT2, BAAT, RARS2, VARS2, PSPH, DHFRP1, LRRC47, RIMKLB, AARS2, CARNS1, PTS, PYCR1, ALDH18A1, ENOPH1, PRODH2, QARS1, BCAT1, QDPR, BCAT2, RARS1, BCKDHA, BCKDHB, AASDHPPT, SARS1, BHMT, SRR, MCCC2, SLC39A8, FN3K, BLMH, ALDH8A1, SHMT1, SHMT2, AGXT2, SLC1A3, PYCR3, SLC16A2, SMS, BPHL, TARS1, TAT, TDO2, TH, TPH1, TST, TYR, VARS1, WARS1, CAD, ALDH5A1, CARS2, NARS2, AGMAT, DGLUCY, ASRGL1, TARS2, AARSD1, CARS1, PCBD2, MRI1, ACCS, GPT2, HPDL, DDO, ATCAY, KMO, YARS1, SLC25A12, ALDH4A1, CBS, PSPHP1, KYAT1, GMPS, KYNU, ARHGAP11B, PLOD3, SLC25A21, SLC7A7, GLYATL1, MARS2, ACY1, SLC25A44, GFPT2, NR1H4 |
| GOBP_REGULATION_OF_CELLULAR_AMINO_ACID_METABOLIC_PROCESS | BCKDK, PARK7, CLN3, ACMSD, SIRT4, SLC7A11, INS, MIR21, ATP2B4, BHMT, ATCAY, SLC7A7, NR1H4 |
| GOBP_POSITIVE_REGULATION_OF_AMINO_ACID_TRANSPORT | SLC38A3, ADORA2A, ABAT, AGT, RAB3GAP1, ARL6IP1, GABBR1, ITGB1, NTSR1, P2RX7, AVP, AVPR1A, AVPR1B, PSEN1, CLTRN, ACE2, SLC6A1, SLC12A2, STXBP1, SYT4, TRH, DTNBP1, KMO |
| GOBP_REGULATION_OF_AMINO_ACID_TRANSMEMBRANE_TRANSPORT | ARL6IP5, SLC43A2, AGT, ARL6IP1, ITGB1, ATP1A2, PSEN1, CLTRN, ACE2, RGS2, RGS4, TNF, SLC43A1, PER2 |
| GOBP_REGULATION_OF_AMINO_ACID_TRANSPORT | ARL6IP5, SLC38A3, HRH3, SLC43A2, ADORA1, ADORA2A, ABAT, AGT, RAB3GAP1, ARL6IP1, GABBR1, GRM2, GRM7, HTR1B, ITGB1, LEP, ATP1A2, NPY5R, NTSR1, P2RX7, AVP, AVPR1A, AVPR1B, PSEN1, CLTRN, ACE2, RGS2, RGS4, SLC6A1, SLC12A2, SNCA, STXBP1, SYT4, TNF, TRH, DTNBP1, SLC43A1, KMO, PER2, SV2A |
| GOBP_NEGATIVE_REGULATION_OF_AMINO_ACID_TRANSPORT | ARL6IP5, HRH3, SLC43A2, ADORA1, ABAT, GABBR1, GRM7, HTR1B, LEP, NPY5R, RGS2, RGS4, TNF, TRH, SLC43A1 |
| GOBP_RESPONSE_TO_AMINO_ACID_STARVATION | CDKN1A, RRAGB, NPRL2, RRAGA, GCN1, KPTN, ATF2, SESN3, KICS2, BMT2, DAP, EIF2S1, FLCN, RNF152, SZT2, LARP1, MTOR, SESN1, FAS, MAP3K5, EIF2AK4, ATF3, ATF4, SH3GLB1, LARS1, SLC38A2, MIOS, IMPACT, ITFG2, PRKD1, MAPK1, MAPK3, MAPK8, EIF2AK2, RRAGD, RRAGC, UCP2, TFEB, WDR59, NPRL3, SEH1L, SESN2, EIF2A, WDR24, MAP1LC3A, BECN1, DAPL1, EIF2AK3, DEPDC5 |

**Supplementary Table 2. Amino acid biosynthetic and transport pathways from GO.**

| Pathways | Organism |
| --- | --- |
| GOBP_ALANINE_TRANSPORT | Homo sapiens |
| GOBP_ARGININE_BIOSYNTHETIC_PROCESS | Homo sapiens |
| GOBP_ASPARTATE_FAMILY_AMINO_ACID_BIOSYNTHETIC_PROCESS | Homo sapiens |
| GOBP_ASPARTATE_TRANSMEMBRANE_TRANSPORT | Homo sapiens |
| GOBP_GLUTAMATE_BIOSYNTHETIC_PROCESS | Homo sapiens |
| GOBP_GLUTAMINE_FAMILY_AMINO_ACID_BIOSYNTHETIC_PROCESS | Homo sapiens |
| GOBP_GLUTAMINE_TRANSPORT | Homo sapiens |
| GOBP_L_ALANINE_TRANSPORT | Homo sapiens |
| GOBP_L_ASPARTATE_TRANSMEMBRANE_TRANSPORT | Homo sapiens |
| GOBP_L_GLUTAMATE_TRANSMEMBRANE_TRANSPORT | Homo sapiens |
| GOBP_L_HISTIDINE_TRANSMEMBRANE_TRANSPORT | Homo sapiens |
| GOBP_L_LYSINE_TRANSMEMBRANE_TRANSPORT | Homo sapiens |
| GOBP_L_PROLINE_BIOSYNTHETIC_PROCESS | Homo sapiens |
| GOBP_L_PROLINE_TRANSMEMBRANE_TRANSPORT | Homo sapiens |
| GOBP_L_SERINE_BIOSYNTHETIC_PROCESS | Homo sapiens |
| GOBP_L_SERINE_TRANSPORT | Homo sapiens |
| GOBP_LEUCINE_TRANSPORT | Homo sapiens |
| GOBP_PROLINE_TRANSMEMBRANE_TRANSPORT | Homo sapiens |
| GOBP_PROLINE_TRANSPORT | Homo sapiens |
| GOBP_SERINE_FAMILY_AMINO_ACID_BIOSYNTHETIC_PROCESS | Homo sapiens |
| GOBP_SERINE_TRANSPORT | Homo sapiens |
| GOBP_SULFUR_AMINO_ACID_BIOSYNTHETIC_PROCESS | Homo sapiens |
| GOBP_SULFUR_AMINO_ACID_TRANSPORT | Homo sapiens |
| GOBP_TRYPTOPHAN_TRANSPORT | Homo sapiens |

**Supplementary Table 3. Amino acid catabolic and metabolic pathways from GO.**

| Pathways | Organism |
| --- | --- |
| GOBP_ALANINE_CATABOLIC_PROCESS | Homo sapiens |
| GOBP_ARGININE_CATABOLIC_PROCESS | Homo sapiens |
| GOBP_ARGININE_METABOLIC_PROCESS | Homo sapiens |
| GOBP_ASPARAGINE_METABOLIC_PROCESS | Homo sapiens |
| GOBP_ASPARTATE_FAMILY_AMINO_ACID_CATABOLIC_PROCESS | Homo sapiens |
| GOBP_ASPARTATE_FAMILY_AMINO_ACID_METABOLIC_PROCESS | Homo sapiens |
| GOBP_ASPARTATE_METABOLIC_PROCESS | Homo sapiens |
| GOBP_CYSTEINE_CATABOLIC_PROCESS | Homo sapiens |
| GOBP_CYSTEINE_METABOLIC_PROCESS | Homo sapiens |
| GLUTAMATE_CATABOLIC_PROCESS | Homo sapiens |
| GLUTAMATE_METABOLIC_PROCESS | Homo sapiens |
| GOBP_GLUTAMINE_FAMILY_AMINO_ACID_CATABOLIC_PROCESS | Homo sapiens |
| GOBP_GLUTAMINE_FAMILY_AMINO_ACID_METABOLIC_PROCESS | Homo sapiens |
| GOBP_GLUTAMINE_METABOLIC_PROCESS | Homo sapiens |
| GOBP_HISTIDINE_CATABOLIC_PROCESS | Homo sapiens |
| GOBP_HISTIDINE_METABOLIC_PROCESS | Homo sapiens |
| GOBP_HOMOCYSTEINE_METABOLIC_PROCESS | Homo sapiens |
| GOBP_ISOLEUCINE_METABOLIC_PROCESS | Homo sapiens |
| GOBP_L_PHENYLALANINE_METABOLIC_PROCESS | Homo sapiens |
| GOBP_L_SERINE_CATABOLIC_PROCESS | Homo sapiens |
| GOBP_L_SERINE_METABOLIC_PROCESS | Homo sapiens |
| GOBP_LEUCINE_CATABOLIC_PROCESS | Homo sapiens |
| GOBP_LEUCINE_METABOLIC_PROCESS | Homo sapiens |
| GOBP_LYSINE_METABOLIC_PROCESS | Homo sapiens |
| GOBP_PROLINE_METABOLIC_PROCESS | Homo sapiens |
| GOBP_SERINE_FAMILY_AMINO_ACID_CATABOLIC_PROCESS | Homo sapiens |
| GOBP_SERINE_FAMILY_AMINO_ACID_METABOLIC_PROCESS | Homo sapiens |
| GOBP_SULFUR_AMINO_ACID_CATABOLIC_PROCESS | Homo sapiens |
| GOBP_SULFUR_AMINO_ACID_METABOLIC_PROCESS | Homo sapiens |
| GOBP_THREONINE_CATABOLIC_PROCESS | Homo sapiens |
| GOBP_THREONINE_METABOLIC_PROCESS | Homo sapiens |
| GOBP_TRYPTOPHAN_CATABOLIC_PROCESS | Homo sapiens |
| GOBP_TRYPTOPHAN_METABOLIC_PROCESS | Homo sapiens |
| GOBP_TYROSINE_CATABOLIC_PROCESS | Homo sapiens |
| GOBP_TYROSINE_METABOLIC_PROCESS | Homo sapiens |
| GOBP_VALINE_METABOLIC_PROCESS | Homo sapiens |

| **Supplementary Table 4. The 91 survival-related amino acid metabolism genes by univariate Cox regression analysis.** | | | | | | |
| --- | --- | --- | --- | --- | --- | --- |
| Tag | HR | Lower | Upper | Likelihood | logrank | Wald |
| BCKDK | 2.549918627 | 1.707469628 | 3.808023812 | 5.57113E-06 | 4.47721E-06 | 4.77083E-06 |
| SERINC5 | 0.606609327 | 0.486776238 | 0.755942561 | 8.25334E-06 | 8.70886E-06 | 8.52008E-06 |
| ETFB | 3.26705912 | 1.923463007 | 5.549197078 | 5.5555E-06 | 9.5785E-06 | 1.18659E-05 |
| UCP2 | 1.97089873 | 1.449605083 | 2.679655204 | 9.51163E-06 | 1.24074E-05 | 1.49892E-05 |
| TFEB | 1.521118386 | 1.248469211 | 1.853310537 | 2.3482E-05 | 2.4504E-05 | 3.15552E-05 |
| SFXN3 | 1.586773785 | 1.238102392 | 2.03363717 | 0.000113772 | 0.000283764 | 0.000265252 |
| ARL6IP5 | 2.032967023 | 1.381650165 | 2.991317933 | 0.000243248 | 0.000314017 | 0.000317574 |
| ECHS1 | 2.144848448 | 1.373600355 | 3.34913634 | 0.000632008 | 0.000818649 | 0.000790522 |
| IL4I1 | 1.389725955 | 1.140549665 | 1.693339877 | 0.002466453 | 0.000935473 | 0.001096888 |
| PCBD2 | 0.46887983 | 0.296308488 | 0.741957466 | 0.002163844 | 0.001146814 | 0.001218234 |
| TRH | 0.873856939 | 0.80461828 | 0.949053693 | 0.000875682 | 0.001200846 | 0.00136719 |
| CTH | 0.627192516 | 0.471046213 | 0.835099489 | 0.001418299 | 0.001287079 | 0.00140498 |
| SDSL | 1.441500767 | 1.14953506 | 1.80762165 | 0.001365039 | 0.001409067 | 0.001541357 |
| VARS1 | 1.8914443 | 1.272090088 | 2.8123492 | 0.001582386 | 0.001633915 | 0.001638001 |
| SLC66A1 | 1.6987269 | 1.222716726 | 2.360050386 | 0.001610772 | 0.001637315 | 0.001585554 |
| HSD17B10 | 2.066918005 | 1.31505001 | 3.248659754 | 0.001148711 | 0.001647965 | 0.001649226 |
| SESN1 | 1.513857865 | 1.170249381 | 1.958356632 | 0.001783311 | 0.001666707 | 0.001594668 |
| RRAGA | 2.790630762 | 1.467646238 | 5.306196991 | 0.001414366 | 0.001755617 | 0.001747272 |
| ASPG | 0.139906829 | 0.032374969 | 0.604600448 | 2.22769E-05 | 0.001910082 | 0.00844337 |
| TPP2 | 0.538806637 | 0.362062753 | 0.801829489 | 0.00278266 | 0.002396392 | 0.002297165 |
| PYCR2 | 2.151475827 | 1.303720202 | 3.550492066 | 0.002554815 | 0.002590487 | 0.002720432 |
| ADSS2 | 0.488225815 | 0.307182945 | 0.775969012 | 0.004017748 | 0.002625461 | 0.00242212 |
| SEPHS2 | 2.544731905 | 1.392690247 | 4.649749273 | 0.001620828 | 0.002730438 | 0.002389602 |
| PDPN | 0.473728973 | 0.242689005 | 0.924719025 | 5.24415E-05 | 0.002929716 | 0.028574519 |
| MFSD12 | 1.828798489 | 1.218173958 | 2.745506 | 0.003467519 | 0.003542044 | 0.003591606 |
| PARK7 | 2.352089687 | 1.309137307 | 4.225932501 | 0.003353538 | 0.004485219 | 0.004223001 |
| GFPT1 | 0.450875512 | 0.257046985 | 0.790862137 | 0.006212379 | 0.00550124 | 0.005463815 |
| HNMT | 1.19042442 | 1.050732978 | 1.348687373 | 0.007289975 | 0.005661823 | 0.00619969 |
| SDS | 1.670678945 | 1.160473435 | 2.405197786 | 0.006771021 | 0.005686422 | 0.005772233 |
| PSAT1 | 1.288285791 | 1.075346807 | 1.543390718 | 0.009328948 | 0.005706281 | 0.005995575 |
| GLUD2 | 1.981428794 | 1.208804403 | 3.247886968 | 0.006056649 | 0.006989838 | 0.00668682 |
| AUH | 0.540324332 | 0.342638204 | 0.852066058 | 0.009049128 | 0.007814646 | 0.008077181 |
| SLC25A29 | 0.746213871 | 0.600357243 | 0.927506328 | 0.008614656 | 0.008086187 | 0.008335801 |
| PCBD1 | 1.571410042 | 1.119888233 | 2.204978539 | 0.00783413 | 0.009079728 | 0.008920067 |
| SLC6A6 | 1.508111635 | 1.102174794 | 2.063557174 | 0.008734354 | 0.01033923 | 0.010227582 |
| ATCAY | 0.135886391 | 0.029078625 | 0.635006338 | 0.006292271 | 0.010495609 | 0.011172906 |
| ADORA1 | 3.716207435 | 1.333585826 | 10.35568722 | 0.020759769 | 0.011098657 | 0.01205568 |
| SESN2 | 1.454904283 | 1.086500552 | 1.948224021 | 0.010897067 | 0.011526425 | 0.011840454 |
| THAP4 | 1.837160144 | 1.143797168 | 2.950835592 | 0.010320921 | 0.01158366 | 0.011880835 |
| AVPR1B | 0.73212976 | 0.573124535 | 0.935248717 | 0.007008195 | 0.012028668 | 0.012566845 |
| ACCS | 0.774588986 | 0.63357256 | 0.946991924 | 0.0123966 | 0.012416515 | 0.012732538 |
| PHGDH | 1.244383959 | 1.04723708 | 1.478644586 | 0.01049545 | 0.01249497 | 0.012975868 |
| SLC43A2 | 1.224813657 | 1.041585942 | 1.440273369 | 0.014016741 | 0.013660421 | 0.014175759 |
| GOT1 | 1.790050412 | 1.126292607 | 2.844980475 | 0.011863876 | 0.01405586 | 0.013774811 |
| LRRC47 | 1.983732014 | 1.147242821 | 3.430130599 | 0.013411788 | 0.014724581 | 0.014222649 |
| GLYAT | 6.910417638 | 1.445783112 | 33.02976188 | 0.030200815 | 0.015402312 | 0.015442316 |
| SFXN1 | 1.432304245 | 1.0715529 | 1.914506928 | 0.014061045 | 0.015421645 | 0.015234598 |
| SLC25A22 | 1.528042752 | 1.082254169 | 2.157454986 | 0.016375799 | 0.015658613 | 0.015991696 |
| GLUD1 | 1.70883219 | 1.099891021 | 2.654906166 | 0.012984878 | 0.016848101 | 0.017148806 |
| SLC16A2 | 0.75912782 | 0.601914861 | 0.957402923 | 0.008540417 | 0.017086175 | 0.019931607 |
| NOS3 | 1.610153204 | 1.079056615 | 2.402648112 | 0.023517484 | 0.018953037 | 0.019671323 |
| KMO | 1.439119064 | 1.060999409 | 1.951993246 | 0.02126502 | 0.01911803 | 0.019248282 |
| ASS1 | 0.820434584 | 0.692773191 | 0.971620895 | 0.012102102 | 0.019638276 | 0.021814109 |
| GSS | 1.809304653 | 1.096376332 | 2.985820864 | 0.017608198 | 0.020077047 | 0.020342641 |
| SARS1 | 1.973754327 | 1.127169646 | 3.456184396 | 0.012740741 | 0.020525838 | 0.017370505 |
| UROC1 | 0.172418099 | 0.039549594 | 0.751663876 | 0.005694947 | 0.020584376 | 0.01928547 |
| SLC9A3R1 | 1.513188593 | 1.061579601 | 2.156917591 | 0.020357375 | 0.021914643 | 0.021998663 |
| SLC7A11 | 1.375963725 | 1.045281715 | 1.811259245 | 0.029204496 | 0.021990921 | 0.022860898 |
| WARS1 | 1.312906403 | 1.042705723 | 1.65312531 | 0.025003608 | 0.02225369 | 0.020576198 |
| VARS2 | 1.615178987 | 1.066521356 | 2.44608619 | 0.023540955 | 0.023435975 | 0.023568487 |
| HDC | 0.890164562 | 0.803829373 | 0.98577257 | 0.022314888 | 0.024553182 | 0.02540028 |
| SLC25A12 | 1.831171575 | 1.080227084 | 3.104152252 | 0.0227723 | 0.024843058 | 0.024669137 |
| SLC38A1 | 1.180737053 | 1.020276851 | 1.366433029 | 0.022210633 | 0.024969867 | 0.025792213 |
| RRAGD | 0.760123272 | 0.597683408 | 0.966711441 | 0.028680429 | 0.025165337 | 0.025354327 |
| GOT2 | 1.782195387 | 1.072820589 | 2.960625878 | 0.021967963 | 0.025931806 | 0.025654687 |
| BMT2 | 0.6688955 | 0.467972304 | 0.956084764 | 0.033673294 | 0.027060007 | 0.027358378 |
| QDPR | 1.918965331 | 1.069261582 | 3.443898112 | 0.02502865 | 0.028909083 | 0.028932709 |
| LARP1 | 0.569154178 | 0.343232737 | 0.943780833 | 0.03626889 | 0.029042183 | 0.028947501 |
| TARS3 | 0.693469332 | 0.498041946 | 0.965580746 | 0.033133256 | 0.029922364 | 0.03020833 |
| SLC25A21 | 0.452352766 | 0.216338179 | 0.945847958 | 0.013875856 | 0.03015757 | 0.035039939 |
| CARS1 | 1.922132781 | 1.075545624 | 3.435088523 | 0.020773698 | 0.030874818 | 0.027397253 |
| MARS1 | 1.90625298 | 1.064044963 | 3.415081646 | 0.023766083 | 0.031333863 | 0.030110166 |
| SLC38A10 | 1.484992067 | 1.031148343 | 2.138587967 | 0.032913909 | 0.033459882 | 0.03360412 |
| SHMT1 | 1.563857137 | 1.034565646 | 2.363938097 | 0.034652959 | 0.033588724 | 0.033908154 |
| ADHFE1 | 1.356196822 | 1.021069419 | 1.801317115 | 0.033297489 | 0.03495419 | 0.035383366 |
| MAPK3 | 1.53285444 | 1.028896863 | 2.283652346 | 0.035113694 | 0.035282523 | 0.035726778 |
| SLC1A4 | 0.724647402 | 0.536401155 | 0.978957357 | 0.039013702 | 0.035741808 | 0.035857802 |
| PEPD | 1.417755561 | 1.018036308 | 1.97441959 | 0.033165697 | 0.039628102 | 0.038852311 |
| BCKDHB | 0.678534954 | 0.468058538 | 0.983658339 | 0.047064083 | 0.040212069 | 0.040665099 |
| CLN8 | 1.409448652 | 1.01151407 | 1.963932645 | 0.04419838 | 0.041994586 | 0.042601101 |
| HPDL | 1.390615062 | 1.011495797 | 1.911832217 | 0.04514408 | 0.042542345 | 0.042321666 |
| KYNU | 1.192771733 | 1.003935576 | 1.417127197 | 0.041629907 | 0.043758544 | 0.045002509 |
| SLC1A5 | 1.451218342 | 1.009148887 | 2.086941485 | 0.044859813 | 0.045059573 | 0.044526812 |
| TDH | 1.888344404 | 1.011379675 | 3.525723009 | 0.057748753 | 0.045257474 | 0.045990181 |
| DPYD | 1.19042639 | 1.003150788 | 1.412663985 | 0.044195673 | 0.045706431 | 0.045935454 |
| SLC38A3 | 0.463338233 | 0.212690332 | 1.009365663 | 0.021535009 | 0.045818464 | 0.052806714 |
| NPRL2 | 1.607436146 | 1.007642945 | 2.564252523 | 0.042458246 | 0.046205031 | 0.046380157 |
| GCLC | 1.403527938 | 1.006034397 | 1.958074872 | 0.047888057 | 0.046461159 | 0.046001749 |
| SCLY | 0.611589414 | 0.377657856 | 0.990424547 | 0.052490985 | 0.04662748 | 0.045599425 |
| NPRL3 | 1.48309757 | 1.001038652 | 2.197296176 | 0.050841669 | 0.048941815 | 0.049398036 |
| NARS2 | 0.658198023 | 0.433565887 | 0.999212924 | 0.054593202 | 0.049460373 | 0.04956945 |

| **Supplementary Table 5. Prognostic genes generated by LASSO Cox analysis.** | |
| --- | --- |
| Gene | Coef |
| TRH | -0.064359611 |
| HNMT | -0.00548324 |
| TFEB | 0.586901864 |
| SDSL | 0.003986443 |
| SLC43A2 | -0.336453375 |
| SFXN3 | 0.222336255 |
